# Probiotics and their fermented feed: multi-dimensional effects and mechanistic insights on pork quality

**DOI:** 10.1186/s40104-025-01327-1

**Published:** 2026-01-22

**Authors:** Xien Xiang, Yanbing Zhou, Peiran Cai, Shiqi Liu, Tizhong Shan

**Affiliations:** 1 College of Animal Sciences, Zhejiang University, Hangzhou, China; 2 The Key Laboratory of Molecular Animal Nutrition, Ministry of Education, Hangzhou, China; 3 Zhejiang Key Laboratory of Nutrition and Breeding for High-quality Animal Products, Hangzhou, China

**Keywords:** Animal nutrition, Antioxidant activity, Fermented feed, Gut microbiota, Intramuscular fat, Meat quality, Muscle fiber

## Abstract

Pork is rich in various nutrients and serves as a pivotal source of protein in the human diet, accounting for a substantial portion of worldwide meat consumption. With the rapid development of the national economy and the improvement of people’s living standards, the demand for high-quality meat is continuously increasing. However, the production of high-quality pork still faces critical challenges. At present, improving pork quality through probiotics and their fermented feed has become a hot topic of interest and concern. Fermented feed can enhance pork quality by improving meat color, drip loss, tenderness, flavor, intramuscular fat (IMF) content, and nutritional value. In this review, we summarized and discussed the recent advances in fermentation strains, fermentation technology, fermented feed characteristics, and their effects and regulatory mechanisms on pork quality, aiming to provide a theoretical foundation and technical insights for the application of fermented feed in the production of high-quality pork.

**Graphical Abstract:**

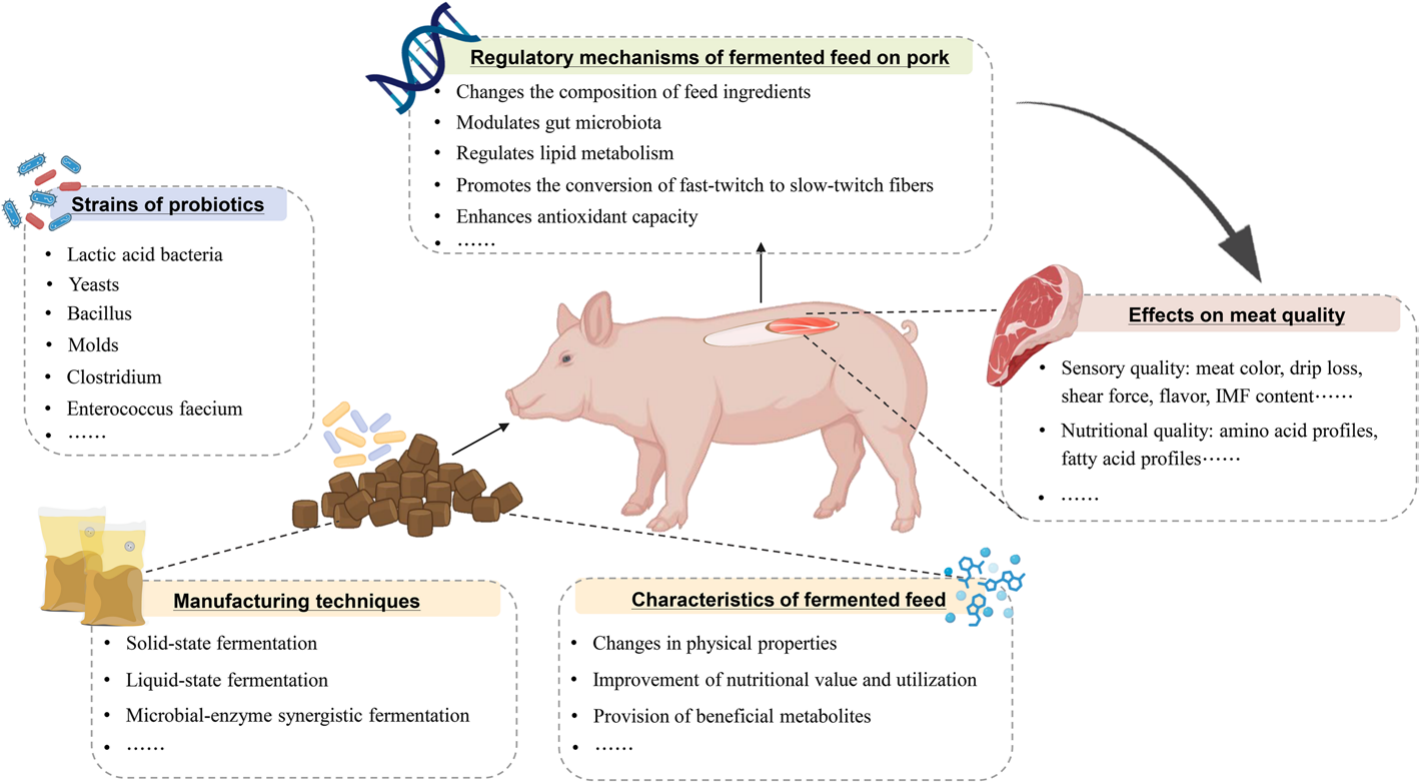

## Introduction

As a nutritionally dense food source, pork plays a vital role in the human diet, providing not only high biological value protein but also essential micronutrients, including B vitamins, iron, and zinc. According to global consumption data, pork constitutes a substantial proportion of total meat consumption [1]. Concurrently, economic growth and rising disposable incomes have led to shifting consumption patterns, with an increasing preference for premium pork products characterized by superior safety, taste, and nutritional value. This growing market preference underscores the great importance of sustained research and methods aimed at improving pork quality.

Pork quality is commonly evaluated based on two principal dimensions: sensory quality and nutritional quality. The sensory quality of pork, which encompasses attributes such as tenderness, flavor, juiciness, and overall acceptability, is largely determined by a range of technological properties. These key determinants include meat color, pH value, drip loss, shear force, intramuscular fat (IMF) content, and marbling score. Meanwhile, nutritional quality is primarily characterized by amino acid (AA) and fatty acid profiles. These quality traits are determined by a complex interplay of intrinsic and extrinsic factors, including genotype, sex, dietary composition, rearing conditions, and slaughter processing parameters [2]. For instance, Liu et al. [3] conducted a comparative study showing that Bama mini-pigs exhibited slower growth rates and reduced carcass yields but superior pork quality compared to Landrace pigs. Similarly, Chen and Sui [4] reported that crossbreeding between wild pigs and domestic breeds can effectively improve meat quality parameters. Furthermore, husbandry management plays a significant role in determining the meat quality. Qi et al. [5] demonstrated that free-range rearing increased the concentrations of flavor amino acids (FAA) and inosine monophosphate (IMP) in muscle, and that extending the feeding cycle promoted fat deposition. In addition to genetic and management factors, nutritional regulation is also an important strategy for optimizing pork quality [6]. Recent studies have highlighted the efficacy of specific dietary interventions in modulating meat quality. Zhang et al. [7] reported that dietary supplementation of 1% leucine promoted the deposition of beneficial fatty acids in the muscle of Shaziling pigs, presenting a viable approach for producing pork with enhanced nutritional quality. Similarly, Wang et al. [8] demonstrated that conjugated linoleic acid (CLA) supplementation improved the lipo-nutritional quality of pork as it effectively enhanced IMF content and lipid metabolism of pigs. Moreover, plant extracts have similarly shown promise as functional feed additives. Liu et al. [9] revealed that mulberry leaf flavonoids positively influenced fatty acid composition in adipose tissue of finishing pigs by increasing n-3 polyunsaturated fatty acids (n-3 PUFA) while reducing the n-6/n-3 PUFA. Guo et al. [10] found that dietary addition of dihydromyricetin improved tenderness, meat color, and AA composition of pork. Collectively, these findings underscore the significant potential of nutritional strategies in improving pork quality, offering promising avenues for producing meat with enhanced sensory and nutritional properties.

At present, fermented feed has become a prominent research focus as a dual-benefit strategy that enhances pork quality while enabling the utilization of both conventional and unconventional feed ingredients. Fermented feed is a type of bioactive feed produced by transforming the raw materials into small bioactive peptides, AA, probiotics, and microbial metabolites [11]. Numerous studies have documented its multifaceted benefits in livestock production. For instance, fermented feed enhances nutrient digestibility and absorption, which greatly improves animals’ growth performance while mitigating environmental impacts associated with livestock production [11, 12]. However, some studies report inconsistent results on growth performance, indicating that fermented feed had negligible impacts on growth performance [13]. These discrepancies are potentially attributable to the variations in experiment design, including animal age, substrate composition, probiotic strains, and addition levels [14]. Moreover, fermented feed demonstrates immunomodulatory and antioxidant properties [15, 16]. Many studies have reported that fermented feed improves pork quality through two primary evaluation dimensions: sensory attributes and nutritional composition. The enhancement of sensory quality is underpinned by measurable improvements in sensory quality relative parameters, including more desirable meat color [17–20], reduced shear force [17, 18, 21–23], lower drip loss [21, 23–25], and increased IMF content [19, 24, 26, 27]. Additionally, fermented feed enhances the nutritional quality of pork and develops pork with distinctive nutritional characteristics by optimizing the AA and fatty acid profiles [26–31]. In this review, we systematically examine recent advancements concerning fermentation strains, fermentation technology, and the characteristics of fermented feed, while elucidating their effects and underlying regulatory mechanisms on pork quality, aiming to provide a theoretical foundation and technical insights for the application of fermented feed in the production of high-quality pork.

## Strains of probiotics for fermentation

High-quality fermentation strains are of great importance in producing high-quality fermented feed. Common probiotic microorganisms employed in fermentation include lactic acid bacteria (LAB), *Bacillus*, yeasts, and molds.

### Lactic acid bacteria

LAB are a type of bacteria capable of producing quantities of lactic acid during the fermentation of available carbohydrates. Owing to their numerous beneficial properties, LAB have emerged as one of the most extensively used strains in both industrial food production and fermented feed manufacturing. These bacteria serve as natural producers of various metabolites, such as vitamins, essential amino acids (EAA), and functional peptides, many of which are gaining increasing attention for their therapeutic potential [32]. Of particular significance are the demonstrated health benefits to both humans and animals conferred by LAB, including immunomodulation, intestinal communities regulation, serum cholesterol reduction, and decreased risk of tumours [33]. Moreover, specific LAB strains exhibit additional functional properties as probiotics, such as notable antioxidant [34] and anticancer activities [35].

In fermented feed applications, LAB play a crucial role in enhancing microbial safety. Their antimicrobial activity effectively suppresses bacterial and fungal contamination, thereby improving feed stability and extending its storage period. A study by Li et al. [36] demonstrated that alfalfa silage inoculated with bacteriocin-producing LAB strains (*Lactobacillus delbrueckII* and *Lactobacillus plantarum*) showed significant inhibition of yeast and mold growth, along with improved fermentation quality and aerobic stability. Similarly, Londero et al. [37] observed that chicken feed supplemented with whey fermented by viable kefir LAB and yeasts exhibited enhanced resistance to fungal contamination. From what has been discussed above, LAB serve both as natural preservatives through antimicrobial activity and as bioactive agents conferring health benefits. These properties position LAB as important fermentation strains in fermented feed production.

### *Bacillus*

*Bacillus* represents one of the most distinctive Gram-positive bacteria, and it has become a prominent candidate for microbial cell factories in recent years due to its diverse species and clear genetic background [38]. During fermentation, *Bacillus* can secrete extracellular enzymes to promote the degradation of anti-nutritional factors in feed ingredients. Xu et al. [39] elucidated that faba bean meal fermented with *Bacillus pumilus* significantly reduced the concentrations of phytic acid and proanthocyanidin while increasing the contents of phenols and flavonoids. Scanning electron microscopy revealed distinct morphological alterations in the fermented substrate, characterized by irregular and rough cell wall surfaces. This difference may result from the enzymatic degradation of the cell wall by *Bacillus pumilus*-derived extracellular enzymes. Similar results were obtained by Zhang et al. [40], who demonstrated that the endoglucanase secreted by *Bacillus pumilus* facilitated the degradation of cellulose in *Moringa oleifera*, consequently promoting the nutrient liberation from *Moringa oleifera* leaf meal. These findings suggest that fermentation with *Bacillus* appears to be a promising method for enhancing feed nutritional quality through degrading anti-nutritional factors and enhancing nutrient bioavailability. Furthermore, certain *Bacillus* strains produce bioactive metabolites with potential therapeutic applications. For example, *Bacillus subtilis* can release 2-hydroxy-4-methylpentanoic acid (HMP), a novel microbial metabolite that significantly improves intestinal barrier function [41]. This discovery highlights the therapeutic potential of probiotic-derived metabolites as novel agents for inflammatory bowel disease (IBD) and other disorders characterized by intestinal barrier dysfunction.

### Yeast

Yeast is extensively used in fermented feed production due to its functional and nutritional benefits. The cell walls of yeast contain substantial quantities of β-glucan and mannan oligosaccharides, which are bioactive compounds known to protect the host from mycotoxin damage [42, 43]. Beyond its protective role, yeast enhances the functional characteristics of fermented feed by producing enzymes and metabolites, as well as synergizing with other microbial communities [43, 44]. *Saccharomyces cerevisiae*, one of the most widely employed species of yeast, effectively reduces phytate and other anti-nutritional factors in feed substrates. Feeding broilers with *Saccharomyces cerevisiae* fermented feed enhances their growth performance while simultaneously promoting bone mineralization in broilers [45–47].

Furthermore, *Saccharomyces cerevisia*e is widely employed as a feed additive to boost productivity and immune function in livestock [48]. Several studies have indicated that *Saccharomyces cerevisia*e culture contains valuable compounds, such as bacterial protein, yeast metabolites, and other beneficial components. A study performed by Vailati-Riboni et al. [49] revealed that *Saccharomyces cerevisiae* fermentation products activated the cellular defense mechanisms, enhancing mammary gland cytoprotection against inflammatory damage while maintaining tissue integrity and health in mid-lactation dairy cows challenged with *Streptococcus uberis* mastitis. Another study also exhibited the health benefits of *Saccharomyces cerevisiae* culture, demonstrating that *Saccharomyces cerevisiae* culture fluid could improve dairy cows' feed intake, milk quality, and energy balance during heat stress [50].

### Molds

Solid-state fermentation mediated by *Aspergillus oryzae* and *Aspergillus niger* effectively decreases the contents of tannin in sorghum and favors the release of phenolic compounds. This bioprocessing approach is considered a promising method for improving the bioavailability of nutrients in unconventional feed ingredients [51]. *Aspergillus* spp. can be used to produce industrial lipases. The production process generally involves using carbohydrates and lipids as carbon sources to culture microorganisms and induce the production of lipases. The production efficiency and catalytic performance of these lipases are modulated by multiple physicochemical parameters, including fermentation methods, medium components, inducer concentrations, pH, temperature, and so forth [52–54].

## Fermentation technology

The production of fermented feed is a dynamic process that involves various factors, such as microorganisms, substrate composition, and process parameters. In the process of manufacturing fermented feed, interactions between the microbial strains, raw material properties, processing techniques, and fermentation parameters determine the final fermentation products quality [55]. Various fermentation techniques are employed in fermented feed production, which can be systematically classified according to three key criteria: (1) moisture content (solid-state fermentation (SSF) or liquid-state fermentation), (2) microbial composition (single-strain, mixed-strain, or microbial-enzyme synergistic fermentation), and (3) oxygen requirements (anaerobic, aerobic, or facultative fermentation). The present review focuses on three principal methods: SSF, liquid-state fermentation, and microbial-enzyme synergistic fermentation.

### Solid-state fermentation

SSF refers to the fermentation performed on non-soluble substances that serve as both physical support and a source of nutrients without a free-flowing liquid medium [56]. This method is generally used to produce fermented dry feed that can be incorporated into basal diets either as whole grains or in processed forms (crushed or powdered) [57]. Over the past decades, extensive in vivo and in vitro investigations have substantiated SSF’s immense potential in practical applications [58, 59]. SSF provides bulk beneficial chemicals and enzymes, including protease [60], amylase [61, 62], lipase [63], cellulase, and xylanases [64]. After SSF, the nutritional quality, bioavailability, and palatability of feed have been enhanced. Consequently, researchers have proposed that SSF is suitable for the resource utilization of agro-industrial residues as it can transform low-value agricultural byproducts and produce value-added products [58, 59]. Furthermore, SSF greatly reduces the content of zearalenone in mycotoxin-contaminated corn and corn gluten meal, appearing to be a promising detoxification method [65].

To the best of our knowledge, SSF is an economically viable method that requires simple techniques and less energy. After SSF, the feed will develop a sour taste, which enhances the palatability of the feed and consequently increases animals’ feed intake. Nevertheless, several technical limitations warrant consideration. Firstly, the low moisture content inherent to SSF systems restricts nutrient diffusion and metabolites transport while adversely affecting the activity of enzymes [55]. Meanwhile, low moisture in SSF environments limits microbial diversity, with fermentation being predominantly mediated by specific microorganisms, mainly *Lactobacillus*, *Bacillus*, yeasts, and molds [55, 56]. Additionally, the lack of standardized quality assessment for fermented products poses challenges for quality control and hinders the further development of SSF. Overall, SSF is an effective, economically feasible strategy for enhancing the nutritional and functional characteristics of feed. Its widespread application in husbandry has been shown to promote animal growth performance, although further standardization and optimization of the process are required to address existing limitations.

### Liquid-state fermentation

Liquid-state fermentation is defined as the process involving the mixture of dry feed components and premixes with either water or liquid food industry co-products, followed by controlled fermentation under specific temperature conditions until steady-state equilibrium is achieved [66, 67]. During the initial phase, the levels of LAB, yeasts, and lactic acid in the fermentation substrate are very low, the pH is high, and, more importantly, with the blooming of enterobacteria. By the second phase, in which a steady state is reached, the opposite characteristics are observed: high levels of LAB, yeasts, and lactic acid, low pH, and low enterobacteria count [68]. LAB constitutes the predominant microbial drivers in fermented liquid feed production. The concentration of LAB, whether naturally or supplemented, directly determines the rate of lactic acid production. The faster lactic acid is produced, the faster the drop in pH and the faster pathogenic bacteria can be reduced, thus further exerting beneficial effects on gastrointestinal ecology [67]. The role of yeasts remains controversial: while demonstrating its enterobacteria-binding capacity that may inhibit the binding of these bacteria to gut epithelium, excessive yeast proliferation produces acetic acid, ethanol, and amylic alcohols that compromise feed palatability as well as reducing dry matter and energy content [69]. The negative effect resulting from excessive yeast growth can be mitigated by weak acid supplementation, such as formic acid, potassium sorbate, and benzoic acid. Plumed-Ferrer and von Wright [70] found that formic acid and potassium sorbate inhibited yeast proliferation across all media tested while maintaining LAB viability, thereby substantially enhancing the quality of fermented liquid products.

Fermented liquid feed can be produced by fermenting complete feed or by separate fermentation of cereal components, followed by mixing with other ingredients. Although complete feed fermentation offers operational simplicity, it may result in a loss of nutrients such as vitamins and AA [71]. Comparatively, cereal liquid feed fermentation appears to be a promising strategy, as it minimizes microbial decarboxylation of AA while simultaneously enhancing feed palatability. O’Meara et al. [72] have revealed that feeding grow-finisher pigs with fermented cereal liquid feed presented superior growth performance and feed conversion efficiency compared with feeding whole diet liquid feed. Similar results were observed in piglets [73]. Therefore, partial fermentation strategies have gained increasing attention in the production of fermented liquid feed [74–76].

Fermented liquid feed is often used in swine production, especially for piglets, by simultaneously providing water and feed, thereby buffering the transition of weaned piglets from liquid to dry feed [77]. However, its application in poultry production remains limited, probably due to concerns regarding feed hygiene, wet litter, and associated animal welfare challenges [78, 79]. Current research indicates that fermented liquid feed supplementation exerts beneficial effects on intestinal function by increasing the populations of beneficial microorganisms and suppressing pathogenic bacteria [76, 80]. Besides its positive impact on gastrointestinal ecology, fermented liquid feed reduces the airborne dust levels in the livestock barn, thereby improving environmental conditions and the health of both animals and workers [69]. On balance, the application of fermented liquid feed has brought many benefits to animal production and seems to be an effective alternative to antibiotics. However, its further development still faces many urgent issues that need to be resolved, such as difficulties in storage and transportation, and the tendency to cause pipeline blockages.

### Microbial-enzyme synergistic fermentation

Microbial-enzyme synergistic fermentation represents an advanced approach that combines microbial activity with enzymatic hydrolysis to optimize the quality of fermented products. This integrated approach demonstrates superior efficacy in both anti-nutritional factors degradation and nutritional value enhancement of various feed substrates. Substantial evidence demonstrates the efficacy of microbial-enzyme synergistic fermentation in utilizing various agro-industrial byproducts. For instance, previous studies have reported that microbial-enzyme synergistic fermentation treatment of rapeseed meal effectively improves the nutritional value of feed and nutrient digestibility [81–83]. Compared with microbial fermentation and enzymatic hydrolysis alone, co-fermentation could be a more effective way to degrade anti-nutritional factors and enhance the utilization of rapeseed meal, better fulfilling the needs of actual production [82, 83]. Similar results have also been confirmed in defatted rice bran [59], rice straw [84], maize cob [85], potato hash [86], and palm kernel cake [87]. Taken together, more and more researchers attach great significance to microbial-enzyme synergistic fermentation, demonstrating its potential in processing unconventional feed resources. It is an effective way to mitigate the global feed shortages and reduce feed costs.

## Characteristics of fermented feed

**Fig. 1:**
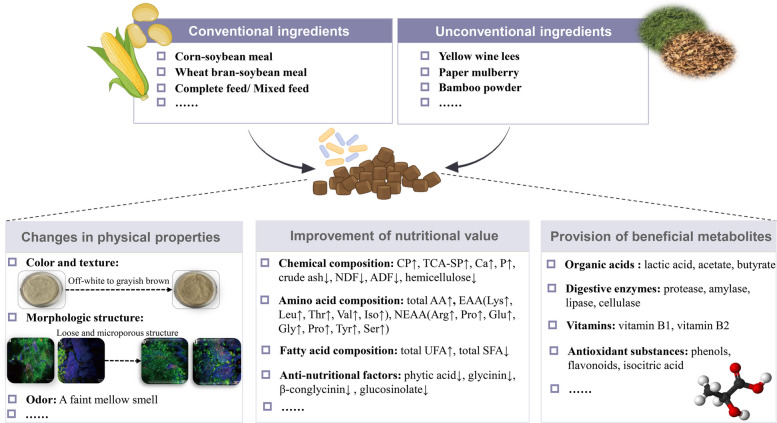
Characteristics of fermented feed. CP: crude protein; TCA-SP: trichloroacetic acid soluble protein; NDF: neutral detergent fiber; ADF: acid detergent fiber; AA: amino acids; EAA: essential amino acids; NEAA: nonessential amino acids; UFA: unsaturated fatty acids; SFA: saturated fatty acids. The image of feed appearance is reprinted from Xu et al. [39], with permission from Elsevier. The image of the scanning electron microscope is reprinted from Su et al. [93], with permission from Elsevier

Microbial fermentation induces significant beneficial modifications in feed ingredients, including physical alterations in odor and microstructure, enhanced nutritional value and digestibility, and the production of bioactive metabolites (Fig. 1). Furthermore, the degree of these improvements exhibits considerable variation depending on the starters employed and the specific fermentation conditions applied to different feed substrates (Table 1).

**Table 1:** Effects of microbial fermentation on the chemical composition of feed

Substrate	Starter culture	Fermentation conditions	Results	References
Corn-soybean meal	*Lactobacillus plantarum*, *Bacillus subtilis*, *Pediococcus pentosaceus*, and *Bacillus coagulans*	Liquid-state fermentation at 30 °C for 3–5 d	Water↓, organic matter↑, CP↑, total AA↑, α-conglycinin↓, β-conglycinin↓, soybean trypsin inhibitor↓	[88]
Corn-soybean meal	*Enterococcus faecium* and *Bacillus subtilis*	Fermented at room temperature and 40% humidity for 72 h	DM↑, CP↑, lactic acid↑, NDF↓, ADF↓, hemicellulose↓, total AA↑, MUFA↑, PUFA↑, SFA↓	[17]
Corn-soybean meal	*Bacillus subtilis*	Fermented at 37 °C and 40% humidity for 24 h	DM↑, CP↑, Energy↑, EE↑, Ash↑	[89]
Corn-soybean meal	*Saccharomyces cerevisiae*,* Bacillus subtilis,* and* Lactobacillus reuteri*	Solid-state fermentation, fermented at 25–35 °C for at least 96 h	DM↓, CP↑, EAA (Leu↑, Val↑, Iso↑, Phe↑, Lys↑, His↑), NEAA (Asp↑, Ser↑, Glu↑, Gly↑, Ala↑, Cys↑, Tyr↑, Pro↑)	[21]
Wheat bran-soybean meal	*Bacillus subtilis* and* Pediococcus pentosaceus*	Fermented at 37 °C and 40% humidity for 24 h	Lactic acid↑, CP↑, Ca↑, P↑, EE↓, UFA(C18:1n-9c↑, C18:2n-6c↑, C18:2n-3↑), TCA-SP↑, carnosine↑, glycinin↓, β-conglycinin↓, NDF↓, hemicellulose↓	[24]
Corn-soybean meal	*Lactobacillus*,* Clostridium*, and* Bifidobacteria*	Fermented at 27–32 °C for 36 h	DM↓, CP↑, EE↓, Ash↓, NDF↓	[90]
Rapeseed meal-wheat bran	*Aspergillus niger*	Solid-state fermentation at 34 °C for 72 h	CP↑, small peptide↑, NDF↓, ADF↓, hemicellulose↓, glucosinolates↓, isothiocyanate↓, oxazolidithione↓, phytic acid↓, EAA(Ile↑, Leu↑, Phe↑, Thr↑), NEAA(Ala↑, Asp↑, Gly↑, Pro↑, Tyr↑)	[91]
Broccoli stem and leaf residues	*Bacillus pumilus* and* Lactobacillus casei*	Fermented at 30 °C and 50% humidity for 15 d	CP↑, CF↑, EE↑, DM↑, total AA↑, EAA (Lys↑), NEAA (Arg↑, Pro↑), TCA-SP↑, lactic acid↑	[92]
Maize cob	*Lactobacillus fermentum, Saccharomyces cerevisiae, Bacillus subtilis* and NSP enzyme	Fermented at 25–30 °C for 5–7 d	CP↑, DM↓, ash↓, NDF↓, ADF↓, EE↓, Ca↑, P↑	[85]
Okara	*Saccharomyces cerevisiae, Bacillus subtilis,* and* Lactobacillus plantarum*	Anaerobic fermented at 35 °C and 60%−70% humidity for 8 d	Water↓, CP↓, EE↓, polysaccharide↑, lactic acid↑, energy↑, total AA↑, EAA (Val↑, Iso↑, Leu↑, Phe↑, Lys↑, Thr↑), NEAA (Asp↑, Ser↑, Glu↑, Gly↑, Ala↑, Cys↑, Tyr↑, Arg↑, Pro↑)	[19]
Carrot by-product	*Lactobacillus plantarum* and* Saccharomyces cerevisiae*	Anaerobic fermentation at 20 °C for 12 d	Water↓, CP↑, CF↓	[22]
Yellow wine lees	*Bacillus subtilis* and* Enterococcus faecium*	Fermented at 30 °C and 35% humidity for 72 h	CP↑, TCA-SP↑, small peptides↑, reducing sugar↑, ADF↓, NDF↓	[27]

*CP* Crude protein, *AA* Amino acids, *DM* Dry matter, *NDF* Neutral detergent fiber, *ADF* Acid detergent fiber, *MUFA* Monounsaturated fatty acids, *PUFA* Polyunsaturated fatty acids, *SFA* Saturated fatty acids, *EE* ether extract, *EAA* Essential amino acids, *NEAA* Nonessential amino acids, *UFA* Unsaturated fatty acids, *TCA-SP* Trichloroacetic acid soluble protein, *CF* Crude fiber

### Changes in physical properties

The physical properties of feed serve as critical quality indicators, encompassing parameters such as pH, color, odor, and texture, which collectively determine processing performance and stability of feed. Xu et al. [39] documented significant alterations in fermented faba bean meal, with color transitioning from off-white to grayish brown and developing a faint, mellow smell. Further observations via scanning electron microscope showed that the cell wall structure appeared irregular and rough shape, making substrates easier to react with enzymes completely. Wang et al. [94] observed an irregular and more microporous structure in fermented soybean meal and corn. Similarly, Tian et al. [19] also suggested that fermented okara had a looser and more microporous structure compared with unfermented okara. Zheng et al. [95] also observed that soybean meal protein had a smaller, cracked structure and large holes as the protease produced by *Bacillus* can destroy the original structure of soybean meal, enhancing the accessibility of digestive enzymes to the protein substrate. Lin et al. [85] reported that after being fermented by probiotics and non-starch polysaccharide (NSP) enzymes, maize cobs exhibited a deeper color and a loose, soft, and moist texture, as well as producing a wine-like and lactic acid scent. However, these findings are inconsistent with observations by Olukomaiya et al. [96], who stated fermented canola meals presented a lighter color compared to unfermented canola meals. The reason was probably due to different feed ingredients fermented by distinct strains, which may exert different effects. Further investigations are required to elucidate the precise mechanisms responsible for these divergent outcomes.

### Improvements of nutritional value and utilization

The presence of anti-nutritional factors in feed ingredients reduces the nutritional value and utilization efficiency of feed, hindering the application of some feed resources to some extent. Microbial fermentation can transform macromolecules into smaller molecules and degrade the anti-nutritional factors, thereby enhancing the nutritional value and utilization efficiency of feed. Substantial evidence demonstrates the efficacy of microbial fermentation in degrading anti-nutritional factors, including glucosinolate [97], phytic acid [93, 98], glycinin and β-conglycinin [94, 95].

Various studies have consistently shown that fermented feed presented higher nutritional and utilization value than unfermented feed. For example, Lin et al. [85] reported significant increases in crude protein (CP), calcium, and phosphorus content in fermented maize cobs, with decreases in dry matter (DM), crude ash, crude fat, neutral detergent fiber (NDF), acid detergent fiber (ADF), and reducing sugar content. Hao et al. [24] observed elevated levels of trichloroacetic acid soluble protein (TCA-SP) and carnosine, alongside decreased concentrations of glycinin, β-conglycinin, NDF, and hemicellulose in fermented corn-soybean meal. These findings are in partial agreement with the previous results of Shi et al. [97], in which the contents of TCA-SP and CP of fermented rapeseed cake were increased.

In addition to improving the chemical composition of feed, an enhancement of AA composition was observed in fermented feed. Sun et al. [92] observed elevated AA concentrations in fermented broccoli stem and leaf residues, with notable increases in Lys, Pro, and Arg. Liu et al. [17] also obtained similar results, indicating an increase in EAA, nonessential amino acids (NEAA), and total AA. Mok et al. [99] showed a near two-fold augmentation in total AA following fermentation, with particularly significant increases in the EAA Leu, Phe, and Glu. In line with the above results, Mukherjee et al. [98] revealed that the total AA and EAA of feed markedly improved after fermentation. What’s more, microbial fermentation optimizes the fatty acid profiles of feed by modulating the ratio of saturated to unsaturated fatty acids (SFA:UFA). Multiple studies have consistently demonstrated a reduction in SFA while increasing the content of UFA in feed after microbial fermentation [17, 24, 99].

### Production of beneficial metabolites

Fermented feed contains a variety of probiotic microorganisms that confer multiple physiological benefits. These microorganisms not only improve the gut microbiota of animals but also produce beneficial bioactive metabolites, including antioxidant compounds, organic acids, digestive enzymes, and vitamins.

Recent research has demonstrated the enhancement of bioactive components after microbial fermentation. Xu et al. [39] elucidated that the phenolic and flavonoid content of faba bean meal was increased after SSF with *Bacillus pumilus*, both of which are known for their antioxidant properties. Correspondingly, Mok et al. [99] suggested that the abundance of isocitric acid increased notably after fermentation with *Bacillus subtilis*, which contains antioxidant properties that can help to combat oxidative stress by decreasing lipid peroxidation and inflammation. Moreover, fermented feed exhibited significant inhibitory effects against *Escherichia coli* and *Salmonella*, attributable to the high concentrations of lactic acid and volatile fatty acids’ antibacterial effects, and the acidic environment suppressing the proliferation of pathogens [88].

## Effects of fermented feed on the quality of pork

**Fig. 2:**
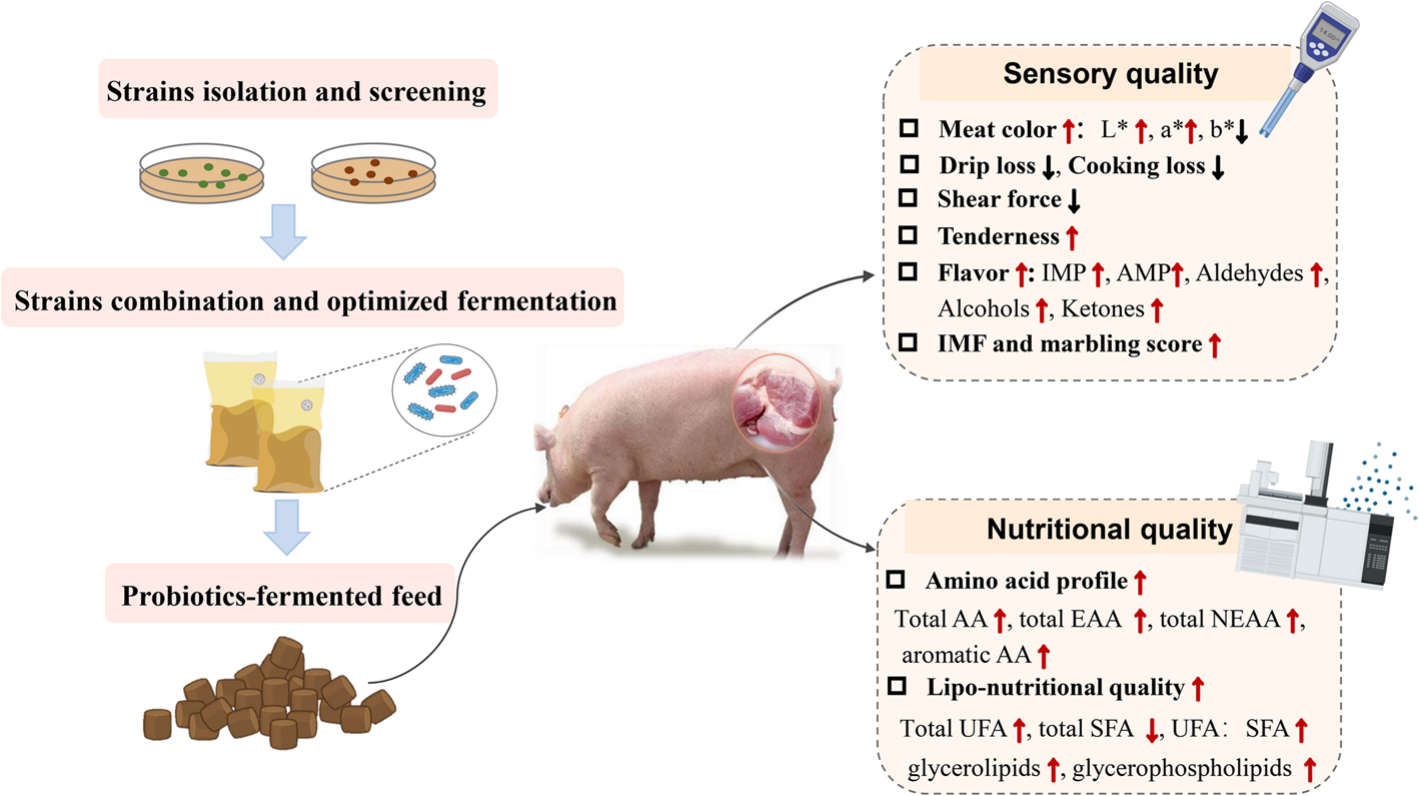
Effects of fermented feed on the meat quality of pork. IMP: inosine monophosphate; AMP: adenosine monophosphate; AA: amino acids; EAA: essential amino acids; NEAA: nonessential amino acids; UFA: unsaturated fatty acids; SFA: saturated fatty acids

Numerous studies have reported that fermented feed improves pork quality through two primary evaluation dimensions: sensory quality and nutritional quality (Fig. 2). The sensory quality of pork is determined by a suite of underlying technological parameters, including meat color, pH value, drip loss, shear force, IMF content, and marbling score (Table 2). Furthermore, fermented feed enhances the nutritional value of pork, particularly the AA and fatty acid profiles of pork (Table 3). These improvements in nutritional quality not only improve the health value of pork but also contribute to extended shelf-life and improved flavor stability.

**Table 2:** Effects of different substrates, starter cultures, and doses on the sensory quality and its relative parameters

Substrate	Starter culture	Dose	Results		References
Corn-soybean meal	*Lactobacillus plantarum*, *Bacillus subtilis*, *Pediococcus pentosaceus*, and *Bacillus coagulans*	10%	Drip loss↓, shear force↓, cooking loss↓, a*↑, b*↓		[88]
Corn-soybean meal	*Enterococcus faecium* and *Bacillus subtilis*	10%	Meat color_45min_↑, meat color_24h_↑, shear force↓, IMF content↑		[17]
Corn-Soybean meal	*Bacillus subtilis*	7.42%	pH_45min_↓, a*_24h_↑		[89]
Corn-soybean meal	*Saccharomyces cerevisiae, Bacillus subtilis, and Lactobacillus reuteri*	100%	Drip loss_48h_↓, shear force↓, tenderness↑, fragrance↑, freshness↑		[21]
Wheat bran-soybean meal	*Bacillus subtilis* and *Pediococcus pentosaceus*	8%	a*↑, marbling score↑, drip loss↓, IMF content↑		[24]
Corn-soybean meal	*Lactobacillus*, *Clostridium,* and *Bifidobacteria*	100%	Drip loss↓, a*↑		[25]
Corn-soybean meal	*Aspergillus oryzae*	8%	Cooking loss↓		[100]
Corn-soybean meal	*Lactobacillus plantarum*	100%	L*↑, shear force↓		[18]
Corn-soybean meal	*Lactobacillus plantarum, Bacillus coagulans, Bacillus subtilis,* and yeast	10%	L*_45min_↓, a*_45min_↑, b*_45min_↓, L*_24__h_↓, a*_24__h_↑, b*_24__h_↓, drip loss↓, shear force↓, marbling scores↑		[101]
Corn-soybean meal	*Bacillus subtilis, Enterococcus faecalis, Saccharomyces cerevisiae,* and* Clostridium butyricum*	100%	L*↓, cooking loss↓		[102]
Corn-wheat bran meal	*——*	15%	IMF content↑, cooking loss↓, pH_45min_↓, meat color_24h_↑, marbling score_24h_↑, a*_24h_↑, b*_24h_↑		[103]
Pine needles	*Aspergillus niger*	2%	pH_24h_↑, L*↓, a*↑, b*↓, shear force↓, marbling scores↑		[104]
Cassava residue meal	*Lactobacillus plantarum*, *Saccharomyces cerevisiae,* and *Bacillus subtilis*	5%	L*↓, a*↑, IMF content↑		[26]
Inonotus obliquus	*——*	8 g/kg	L*_45min_↓, a*_45min_↑, b*_45min_↓, pH_24h_↑, shear force_24h_↓, IMF content↑		[105]
Navel orange pulp	*Lactobacillus*,* Bacillus subtilis* and yeast	10%	L*_24h_↑, drip loss↓		[30]
Broccoli stems and leaf residues	*Bacillus pumilus*, *Enterococcus faecium*, and *Saccharomyces cerevisiae*	10%	Marbling score↑		[106]
Broccoli stems and leaf residues	*Bacillus pumilus* and *Lactobacillus casei*	10%	L*↑, b*↑		[92]
Bamboo powder	*Yersinia lactis* and *Lactobacillus plantarum*	5%	pH_24h_↑, L*↑, a*↑		[20]
Carrot by-product	*Lactobacillus plantarum* and *Saccharomyces cerevisiae*	9%	Shear force↓, L*↓, b*↓		[22]
Mulberry silage or paper mulberry silage	*——*	10%	Marbling score↑ in mulberry silage group; L*↑, water-holding capacity↑, marbling score↑ in paper mulberry group		[107]
Yellow wine lees	*Bacillus subtilis* and* Enterococcus faecium*	8%	IMF content↑, meat color score↑, a*↑		[27]
Banana stem	*——*	50%	Shear force↑		[108]
Maize cob	*Lactobacillus fermentum*,* Saccharomyces cerevisiae*,* Bacillus subtilis*, and NSP enzyme	6%		Meat color score↑, marbling score↑, pH_45min_↑, pH_24h_↑, tenderness↓, drip loss↓	[85]
Tea residue	*Bacillus subtilis*,* Aspergillus niger*, and* Saccharomyces cerevisiae*	15%		pH_45min_↑, pH_24h_↑, L*↓, drip loss↓, shear force↓	[23]
Okara	*Saccharomyces cerevisiae*, *Bacillus subtilis*, and *Lactobacillus plantarum*	Replacement of 44.55% corn, 28% soybean meal, and 100% wheat bran		a*_48h_↑, IMF content↑	[19]
Apple pomace-mixed silage	*——*	5%		Water-holding capacity↓	[109]
Persimmon	*Bacillus subtilis*,* Bifidobacterium pseudolongum*,* Lactobacillus acidophilus*, and yeast	10%		pH↑, shear force↑, drip loss↓, L*↑, b*↓, juiciness↑	[110]
*Flammulina velutipes* by-product	*Lactobacillus plantarum* and* Saccharomyces cerevisiae*	70%		pH↑, cooking loss↓, shear force↓, L*↓, a*↓, b*↓	[111]
Grape pomace	*Saccharomyces boulardII*	30 g/kg		a*↑, b*↑	[112]
Garlic powder	*Weissella koreensis*	4 g/kg		Marbling score↑	[113]
Red ginseng	*Bifidobacterium*	4 g/kg		Drip loss↓	[114]
Apple	*——*	4%		pH_24h_↑, water-holding capacity↑, a*↑, b*↓, tenderness↑, juiciness↑	[115]
Food co-products and wastes	Lactic	100%		Tenderness↑	[116]
Pleurotus ostreats by-product	*——*	3%		pH_24h_↓, water-holding capacity↑, a*↓, b*↓	[117]

*IMF* Intramuscular fat

**Table 3:** Effects of different substrates, starter cultures, and doses on the nutritional quality of pork

Substrate	Starter culture	Dose	Results	References
Corn-soybean meal	*Enterococcus faecium* and *Bacillus subtilis*	10%	Amino acid profiles: total FAA↑, total EAA↑ (Lys↑, Met↑, Iso↑, Phe↑, Thr↑), total NEAA↑ (Ala↑, Asp↑, Glu↑, Arg↑, Ser↑, Tyr↑), total AA↑; Fatty acid profiles: total PUFA↑, PUFA: SFA↑, total n-3 PUFA↑ (C18:3n3↑, C20:3n3↑), total n-6 PUFA↑ (C18:2n6c↑)	[17]
Corn-soybean meal	*Lactobacillus plantarum*, *Candida utilis*, *Bacillus subtilis*, and *Aspergillus niger*	100%	Amino acid profiles: EAA (Lys↑), NEAA (Glu↑); Fatty acid profiles: total UFA↑ (C18:1n9c↑, C18:2n6c↑, C20:4n6↑), total SFA↓ (C18:0↓)	[118]
Wheat bran-soybean meal	*Bacillus subtilis* and *Pediococcus pentosaceus*	8%	Fatty acid profiles: total SFA↓ (C18:0↓), total UFA↑ (C18:1n9c↑, C18:2n6c↑, C18:3n3↑, C20:2↑, C20:4n6↑);	[24]
Corn-soybean meal	*Lactobacillus*, *Clostridium*, and *Bifidobacteria*	100%	Amino acid profiles: NEAA (Asp↑, Glu↑, Ala↑); Fatty acid profiles: UFA (C16:1n7↑, C20:5n3↑), SFA (C17:0↑)	[25]
Corn-soybean meal	*Lactobacillus plantarum*,* Bacillus coagulans*,* Bacillus subtilis*, and yeast	10%	Fatty acid profiles: total SFA↓ (C18:0↓), total MUFA↑ (C18:1n-9↑), total PUFA↑ (C18:2n-6↑, C20:4n-6↑)	[101]
Corn-soybean meal	*Bacillus subtilis*,* Enterococcus faecalis*,* Saccharomyces cerevisiae*, and* Clostridium butyricum*	100%	Amino acid profiles: EAA (His↓), NEAA (Arg↓, Pro↓)	[102]
Corn-wheat bran meal	*——*	15%	Amino acid profiles: Val↑, Cys↑; Fatty acid profiles: C18:3n3↑, n-6/n-3 PUFA↓	[103]
Cassava residue meal	*Lactobacillus plantarum*, *Saccharomyces cerevisiae*, and *Bacillus subtilis*	5%	Fatty acid profiles: total SFA↓ (C20:0↓), SFA/UFA↓	[26]
Inonotus obliquus	*——*	8 g/kg	Amino acid profiles: total EAA↑ (Lys↑, Leu↑, His↑, Phe↑), NEAA (Gly↑, Ala↑), total AA↑, total FAA↑; Fatty acid profiles: total PUFA↑ (C18:2n6↑, C18:3n6↑, C20:2↑, C20:4n6↑, C22:6n3↑), total SFA↓ (C11:0↓, C16:0↓), total n-3 PUFA↑, total n-6 PUFA↑, PUFA/SFA↑, C18:1n9t↑	[105]
Navel orange pulp	*Lactobacillus*,* Bacillus subtilis* and yeast	10%	Fatty acid profiles: total MUFA↑ (C18:1n9↑), total PUFA↑ (C18:2n6↑), total SFA↓ (C18:0↓), UFA:SFA↑	[30]
Mulberry silage	*——*	10%	Fatty acid profiles: total SFA↓ (C18:0↓, C14:0↓), total MUFA↓ (C18:1↓), total PUFA↑ (C20:2↑, C20:4↑, C18:2↑), total n-6 PUFA↑	[107]
Yellow wine lees	*Bacillus subtilis* and* Enterococcus faecium*	8%	Amino acid profiles: EAA (Thr↑, Val↑, Met↓, Ile↓, Leu↑, Phe↑, Lys↑), NEAA (Asp↑, Ser↑, Glu↑, Gly↑, Ala↑)	[27]
Maize cob	*Lactobacillus fermentum*,* Saccharomyces cerevisiae*,* Bacillus subtilis* and NSP enzyme	6%	Fatty acid profiles: total SFA↑, total UFA↑, total MUFA↑, total PUFA↓	[85]
Okara	*Saccharomyces cerevisiae*, *Bacillus subtilis*, and *Lactobacillus plantarum*	Replacement of 44.55% corn, 28% soybean meal, and 100% wheat bran	Amino acid profiles: Thr and Pro in *longissimus thoracis*↑, Thr in *biceps femoris muscle*↑, Thr, Ser, and Asp in *semitendinosus* muscles↑	[19]
Herbs (*Artemisia capillaries* and *Acanthopanax* senticosus)	*Enterococcus faecium*	0.05%	Fatty acid profiles: PUFA/SFA↑, SFA (C14:0↓, C18:0↓), UFA (C18:3n3↑)	[29]
Biogas residue	Distiller’s yeast	10%	Amino acid profiles: total EAA↑ (Thr↑, Met↑, Phe↑, Leu↑, Lys↑, Pro↑), total NEAA↑ (Asp↑, Glu↑, Ser↑, Arg↑, Ala↑, Tyr↑), total AA↑	[31]
Herb combination (pomegranate, *Ginkgo biloba*, and licorice)	*Lactobacillus plantarum* and *Saccharomyces cerevisiae*	0.4%	Fatty acid profiles: total MUFA↑ (C18:1↑), total n-3 PUFA↑ (C18:3n3↑, C20:5n3↑), SFA (15:0↓)	[28]
Apple pomace-mixed silage	*——*	5%	Fatty acid profiles: total PUFA↑ (C18:2↑, C18:3↑), SFA (C16:0↓, C20:0↑), MUFA (C16:1↓, C17:1↓)	[109]
Persimmon	*Bacillus subtilis*,* Bifidobacterium pseudolongum*,* Lactobacillus acidophilus*, and yeast	10%	Fatty acid profiles: total SFA↓ (C16:0↓, C18:0↓), total UFA↑ (C18:1n9↑), UFA/SFA↑	[110]
*Flammulina velutipes* by-product	*Lactobacillus plantarum* and* Saccharomyces cerevisiae*	70%	Fatty acid profiles: UFA (C16:1↑, C20:4n-6↑)	[111]
Grape pomace	*Saccharomyces boulardII*	30 g/kg	Fatty acid profiles: total SFA↓ (C16:0↓, C18:0↓, C20:0↓), total PUFA↑ (C18:2n6↑)	[112]
Feed co-products and wastes	Lactic	100%	Fatty acid profiles: SFA↓, UFA↑, MUFA↑, PUFA↑	[116]
*Pleurotus ostreats* by-product	*——*	3%	Fatty acid profiles: total SFA↓ (C16:0↓, C18:0↓), total UFA↑ (C16:1↑, C18:1n9c↑, C18:2n6c↑, C20:4n6↑), SFA:UFA↓	[117]

*FAA* Flavor amino acids, *EAA* Essential amino acids, *NEAA* Nonessential amino acids, *AA* Amino acids, *PUFA* Polyunsaturated fatty acids, *UFA* Unsaturated fatty acids, *SFA* Saturated fatty acids, *MUFA* Monounsaturated fatty acids

### Effects of fermented feed on the sensory quality and its relative parameters

#### Meat color

Meat color serves as the primary determinant influencing consumer purchasing decisions because it is the key indicator of freshness and wholesomeness than any other quality attributes from a consumer perspective [119]. Consumers are more willing to pay for bright red meat, while dark or pale meat colors are associated with reduced consumer acceptability and increased product rejection [120]. The color of fresh meat depends on the concentration and redox state of myoglobin: deoxymyoglobin presents as dark purple-red, oxymyoglobin is bright red, and metmyoglobin is brown [121]. Meat color is usually represented in the lightness (L* value), redness (a* value), and yellowness (b* value). Pigment concentrations and myoglobin forms accounted for much of the variation in a* value, while b* value was primarily determined by the myoglobin oxidation state [122].

Many researchers attach great importance to the impact of fermented feed on the meat color. For example, Xu et al. [30] indicated that 10% fermented navel orange pulp supplementation improved the meat color by increasing the L* value while reducing the b* value. Liu et al. [17] elucidated that 5% and 10% fermented mixed feed effectively improved the meat color_45min_ and meat color_24h_. Zheng et al. [20] reported that 5% fermented bamboo powder markedly increased L* and a* value, and tended to reduce b* value, making the meat color fresher. Similarly, Chu et al. [110] showed that a dietary fermented persimmon diet had a negligible effect on a* value, but significantly increased the L* value and reduced the b* value. Xie et al. [89] illustrated that a fermented soybean meal-containing diet tended to increase a* value. However, there are some inconsistent findings: Sun et al. [92] found that the L* value and b* value were higher in the *longissimus dorsi* muscle from pigs receiving 10% fermented broccoli residue supplementation. Chu and Park [22] revealed that fermented carrot by-products reduced the L* value and b* value. Fermented feed ameliorating meat color is probably attributable to some metabolites produced during fermentation. Specifically, Organic acids contained in the fermented apple diet boost the secretion of gastric juices, thereby facilitating iron absorption. Subsequently, the absorbed iron can combine with myoglobin, ultimately enhancing meat redness [115].

#### pH value

Meat pH value measured at 45 min (pH_45min_) and 24 h (pH_24h_) post-slaughter serves as a significant index for evaluating the sensory quality of pork. Meat pH value is closely associated with shelf-life and water-holding capacity of pork [28]. Most importantly, changes in pH value can cause alterations in meat color. High pH meat has large diameter muscle fibers due to reduced shrinkage and is accompanied by more transmittance and less light scattering and reflectance, thus presenting a darker color. Low-pH meat, on the other hand, undergoes greater transverse shrinkage of the lattice and is accompanied by greater muscle fiber shrinkage and more light scattering, resulting in a lighter color, probably due to the decrease in pH leading to enhanced protein denaturation and structural changes [123, 124]. According to Moeller et al. [125], the ultimate pH influences consumer perception of pork eating quality, with meat products near pH 5.40 receiving reduced consumer acceptability, while incremental improvements in juiciness, tenderness, and flavor attributes were observed as pH increased toward 6.40 [125]. Interestingly, Ding et al. [23] revealed that fermented tea residue increased pH_45min_ of pork, but as the addition amount increased from 15% to 20%, the pH gradually decreased. These findings were consistent with Lee et al. [115], who showed that a fermented apple diet significantly improved pH_24h_ of *longissimus dorsi* muscle, but as the addition amount increased from 2% to 6%, the pH gradually decreased.

In summary, pH value is a key determinant of meat quality, influencing color, shelf-life, and consumer acceptability. While fermented feed can modulate pH value, the effects are dose-dependent, suggesting an optimal inclusion level for maximizing benefits.

#### Drip loss

Drip loss, together with cooking loss and muscle water loss, is often used to comprehensively assess the water-holding capacity of muscle. Lower water-holding capacity is tightly associated with a loss of nutritional value, and what’s worse is that it leads to drier and harder meat [100]. Some researchers have unequivocally demonstrated that the integration of fermented feed into the diet of pigs can reduce drip loss in pork, thereby enhancing the muscle's capacity to retain moisture. For instance, Xu et al. [30] reported that 10% fermented navel orange pulp effectively reduced the drip loss of pork. Lu et al. [25] observed reduced drip loss of pork fed fermented corn-soybean meal. Qiu et al. [21] showed that dietary supplementation with a fermented diet markedly reduced the drip loss_48h_ of pork. Lin et al. [85] illustrated that dietary 6% and 8% fermented maize cob feed reduced both water loss rate and drip loss of pork. Similar results were obtained by Ding et al. [23] feeding pigs with fermented tea residue. However, there are still some controversial results on the effect of fermented feed on the water-holding capacity of pork. Zheng et al. [20] elucidated that drip loss was linearly and quadratically increased with the fermented bamboo powder addition. Additionally, Fang et al. [109] observed that pigs fed with apple pomace-mixed silage presented a lower water-holding capacity of meat. Given these conflicting results, it remains unclear whether the negative effects stem from the fermented products themselves or the inherent properties of the argo-industrial products used in fermentation. Therefore, further investigations are warranted to elucidate the precise mechanisms by which fermented feed on the water-holding capacity.

#### Shear force and tenderness

Consumers favor pork with lower shear force. As shear force increases, consumer satisfaction decreases in a sizable, significant manner [125]. Several studies have elucidated the beneficial effects of fermented feed supplementation on reducing the shear force of pork. Specifically, Liu et al. [17] figured out that 10% fermented mixed feed supplementation decreased the shear force of pork relative to the control. This finding aligns with the observations of Chu and Park [22], who reported improved shear force following dietary supplementation with fermented carrot by-products. However, it is worth noting that while moderate supplementation demonstrates positive effects, excessive addition of fermented feed may yield adverse outcomes. For example, 2.5% and 5% fermented bamboo powder showed no significant effect on shear force, but as the addition reached 10%, the shear force of pork increased [20]. Ding et al. [23] also observed a decrease in shear force when supplemented with 10% fermented tea residue and an increase in shear force as the addition reached 15% and 20%. Similarly, a 3% fermented persimmon diet reduced the shear force of pork, but when it was more than 5%, the shear force of pork increased [110]. The reason for this effect is still unclear and may be related to the feed ingredients themselves. Tenderness, which reflects meat texture, is negatively correlated with shear force [100]. Dietary supplementation with fermented feed has been shown to effectively reduce shear force, thereby significantly improving tenderness scores in pork [21, 85].

#### Flavor

Fermented feed improves the flavor of pork by enhancing the FAA and volatile flavor substrates. In detail, the content of total aldehydes, (E,E)-2,4-nonadienal, dodecanal, nonanal, and 2-decenal, which are known to contribute to the characteristic flavor of pork, was enhanced after being treated with 10% fermented mixed feed [126]. Flavor metabolomics analysis further revealed that fermented feed elevates the level of diverse flavor-related metabolites, such as alcohols, esters, hydrocarbons, heterocyclic compounds, and ketones [103]. These changes are likely associated with the increases in precursor AAs and UFAs in pork induced by fermented feed, which collectively modulate flavor formation pathways. Additionally, nucleotides, particularly IMP, play a pivotal role in determining the flavor of pork. IMP is widely regarded as a key umami-enhancing nucleotide and indicator for evaluating the taste of meat products. Recent studies by Liu et al. [126] and Xu et al. [30] have consistently demonstrated that fermented feed supplementation effectively increased the content of IMP in pork. Thereby significantly improving the overall flavor characteristics. Taken together, these alternations mentioned above contribute to an improvement in the flavor of pork.

#### IMF content and marbling score

IMF content is widely recognized as a critical determinant of pork eating quality, positively influencing juiciness, flavor, and tenderness of pork. However, consumer preferences regarding fat vary dramatically between and within cultures. A moderate amount of IMF is favorable for many Asian consumers, whereas visible fat is unpopular with Western consumers [127]. A low content of IMF leads to dry and less tasty eating quality [128]. According to Fortin et al. [129], the minimum level of IMF that ensures a good taste is 1.5%. A study conducted by Fernandez et al. [130] suggested that while increased IMF content enhances consumers’ acceptability of pork. Still, the effect is favorable only if the IMF content is below 3.5% because visible fat may trigger consumer rejection. Another study performed in Spanish suggested that the minimum IMF content to ensure a pleasant eating quality is between 2.2% and 3.4% [131]. Taken together, the appropriate IMF content varies from person to person, highlighting the importance of balanced IMF deposition for optimal meat acceptability.

The effects of fermented feed supplementation on porcine IMF deposition exhibit considerable variation across studies, primarily attributable to differences in fermentation substrates, fermentation strains, and dietary inclusion levels. Several studies have reported positive impacts: Jiang et al. [26] found significantly elevated IMF content in Huanjiang mini-pigs fed 5% fermented cassava residue. Liu et al. [17] also observed higher IMF content in 5% and 10% fermented mixed feed groups. Similarly, Tian et al. [19] noted that fermented okara supplementation increased IMF deposition of pigs. However, neutral and adverse effects have been reported in several studies: Hou et al. [132] detected no significant effect on IMF content supplemented with fermented mulberry, while Zheng et al. [20] reported a decrease in IMF content. These contradictory findings underscore the complex nature of fermented feed on IMF deposition in swine.

Positively associated with IMF content, marbling score—a crucial indicator of both meat eating quality and the distribution pattern of fat within pork muscle, is closely correlated with flavor and tenderness. Emerging evidence suggests that fermented feed supplementation can effectively enhance marbling score. Zhao et al. [106] indicated that dietary fermented broccoli residue supplementation improved the marbling scores of *longissimus thoracis*. Niu et al. [107] reported superior marbling scores in experimental groups receiving either mulberry silage or paper mulberry silage supplementation. Lin et al. [85] showed that 6% fermented maize cob feed improved the marbling scores of pork. Similar results were obtained by Yan et al. [113], who revealed that pigs fed on diets supplemented with 2 g/kg and 4 g/kg fermented garlic powder exhibited greater marbling scores. Collectively, these studies provide compelling evidence that fermented feed can positively influence IMF distribution patterns, thereby potentially improving pork quality characteristics.

### Effects of fermented feed on nutritional quality

#### Improves the amino acid profiles

The composition of AA in pork is directly related to the nutritional quality and flavor of meat. Specifically, the types, contents, and relative proportions of AA in pork influence meat quality parameters. EAA primarily determines the quality of meat proteins, while some FAA, such as Ala, Glu, Gly, Asp, and Ser contribute to the umami taste and palatability of pork [118].

Several studies suggested that the AA composition was improved by microbial fermentation, thereby exerting a positive impact on the nutritional quality and flavor of pork. Liu et al. [17] elucidated that dietary inclusion of 5% and 10% fermented mixed feed increased the concentrations of FAA, total EAA, total NEAA, and total AA compared to the control group. Specifically, the contents of EAA (Lys, Met, and Thr) and NEAA (Ala, Asp, Glu, Arg, Ser, and Tyr) were increased with fermented mixed feed supplementation. Tang et al. [118] observed an increase in Glu and Lys of pork fed with fermented complete feed, which positively influenced both nutritional quality and flavor profile. Likewise, Lu et al. [25] indicated that pigs fed fermented corn-soybean meal have a higher content of aromatic AA, such as Asp, Glu, and Ala. Similar beneficial effects have also been observed when feeding pigs with fermented by-products. Zhang et al. [27] revealed that 8% fermented yellow wine lees effectively improved the AA profiles in pork, especially some flavor precursor AA like Asp, Glu, Gly, Thr, Ser, and Ala, were increased markedly. Similarly, Xu et al. [31] found that 5% and 10% fermented biogas residues increased the content of both EAA and NEAA, specifically, the abundance of Asp, Glu, Ser, Thr, Arg, Ala, Tyr, Met, Phe, Leu, Lys, and Pro was enhanced.

Briefly, the composition of AA of pork plays a fundamental role in determining its nutritional quality and flavor characteristics. Studies utilizing various conventional and unconventional ingredients demonstrate that fermented feed optimizes the AA profiles, thereby improving pork quality.

#### Improves the lipo-nutritional quality

Lipo-nutritional quality represents a crucial parameter for assessing the nutritional value of pork, with particular emphasis on fatty acid composition. Pork fatty acids can be divided into SFA, monounsaturated fatty acids (MUFA), and polyunsaturated fatty acids (PUFA). SFA may elevate serum cholesterol levels and increase the risk of type-2 diabetes and cardiovascular diseases [133]. Conversely, UFA may decrease cholesterol levels and confer various health benefits on human health, in particular PUFA, exhibiting anti-inflammatory properties [134]. Consequently, nutritional strategies, including fermented feed supplementation, have been explored to optimize fatty acid profiles by reducing SFA and enhancing UFA. Emerging evidence demonstrates that fermented feed effectively improves the fatty acid profiles of pork. Xu et al. [30] reported that 10% fermented orange navel pulp reduced the content of stearic acid (C18:0), while increasing the contents of oleic acid (C18:1n-9) and linoleic acid (C18:2n-6) in the *longissimus dorsi* muscle. Similarly, Lu et al. [25] observed an increase in palmitoleic acid (C16:1n-7) and eicosapentaenoic acid (C20:5n-3) of pork following fermented feed supplementation. Tang et al. [118] elucidated that fermented corn-soybean meal effectively improved the fatty acid profiles in pork by reducing the SFA (C18:0), and increasing the UFA (C18:1n-9, C18:2n-6, and arachidonic acid (C20:4n-6)). Fang et al. [109] found that apple pomace-mixed silage increased the total content of PUFA in pork. Specifically, the levels of C18:2n-6 and linolenic acid (C18:3n-3) were increased, while the content of palmitic acid (C16:0) was decreased. Lei et al. [29] elucidated that the contents of myristic acid (C14:0) and C18:0 were decreased while the content of C18:3n-3 increased in fermented herbs dietary treatment. Consistent with the findings above, Ahmed et al. [28] also reported that dietary supplementation with fermented herb combinations reduced the concentration of pentadecanoic acid (C15:0) while increasing the concentrations of C18:1n-9, C18:3n-3, and C20:5n-3. Chu et al. [110] revealed that a dietary fermented persimmon diet decreased the contents of C16:0 and C18:0, whereas the content of C18:1n-9 increased. More recently, scientists have attached great importance to the ratio of n-6:n-3PUFA, which has been identified as a significant risk factor in cancers and coronary heart disease [135]. Recent evidence suggests that fermented feed can favorably modulate the n-6:n-3 ratio [28]. As demonstrated by Liu et al. [17], who observed increased n-3 PUFA content, such as C18:3n-3, and dihomo-α-linolenic acid (C20:3n-3) following dietary fermented mixed feed supplementation.

Besides the composition of fatty acids, lipid composition also plays a crucial role in determining pork nutritional quality, which is intricately linked to human health [8, 136]. Previous studies have identified that glycerolipids and glycerophospholipids constitute the predominant lipid classes in porcine *longissimus thoracis* muscle [8]. Among glycerolipids, triglycerides (TAG) and diglycerides represent the major subclasses. TAGs are primarily stored in adipose and muscle tissues and are one of the decisive factors of IMF content [137]. Glycerophospholipids also serve equally vital roles in the formation of pork quality as they play a pivotal part in the Maillard reaction during the heating process, and their oxidative degradation generates aldehydes, ultimately influencing the flavor of pork [138]. A recent study has found that fermented feed supplementation effectively increased the proportion of TAG and glycerophospholipids, greatly improving the lipo-nutritional quality of pork [139].

To sum up, fermented feed supplementation modifies pork’s fatty acid profiles and lipid composition, reducing SFA while enhancing UFA, and what’s more, increases the contents of TAG and glycerophospholipids. These improvements not only enhance pork’s lipo-nutritional quality but also contribute to boosted flavor and human health benefits.

## The regulatory mechanisms of fermented feed on pork quality

### Changes the composition of feed ingredients

After probiotics fermentation, the content of CP, DM, EAA, and NEAA in the feed is improved, thereby enhancing the digestibility and deposition efficiency of the nutrients [19–21, 27, 89]. What’s more, microbial fermentation induces favorable changes in fatty acid profiles, characterized by elevated MUFA and PUFA content concomitant with reduced SFA level in feed, ultimately ameliorating the nutritional quality of pork [17, 24]. Most importantly, the fermentation process promotes the release of nutrients and a myriad of bioactive substances from some unpalatable and insoluble nature. In this way not only reuses the agro-waste in an economical and environmentally friendly way but also exerts beneficial effects on meat quality. For example, fermented okara had a higher content of polysaccharides than unfermented okara, and polysaccharides have been shown to enhance meat quality by regulating the antioxidant capacity and IMF deposition [19, 140]. Similarly, organic acids contained in fermented apple diet promote gastric juice secretion and enhance iron absorption, thus boosting the combination of iron and myoglobin and eventually enhancing the redness of pork [115]. Furthermore, there was also an increase in the contents of antioxidant compounds during fermentation, and the enhancement of antioxidant activities is associated with the improvement of meat color. Optimal fermentation could enhance the release of antioxidant compounds such as phenolic, flavonoids, small peptides with antioxidant activity, and some antioxidant enzymes, thereby boosting the antioxidant activity even by 45%−55% [141, 142]. Another study showed that fermentation of okara with *Bacillus subtilis* increased antioxidant content by 6.4 times [99, 125]. These findings provide crucial insights into the regulation of meat quality.

### Modulates gut microbiota

**Fig. 3:**
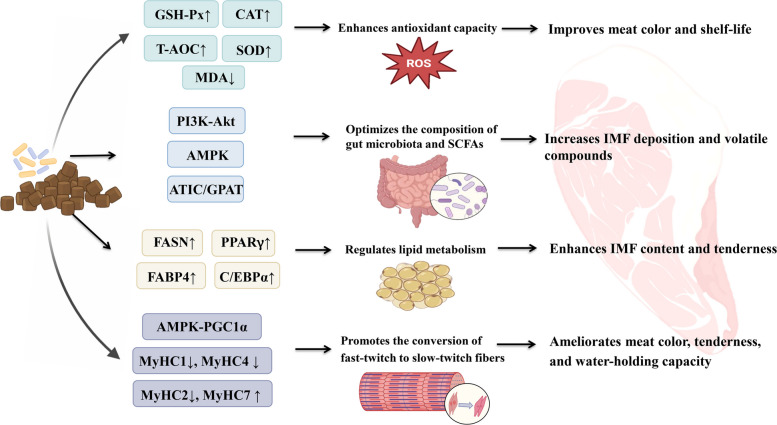
The regulatory mechanisms of fermented feed on pork quality. GSH-Px: glutathione peroxidase; CAT: catalase; T-AOC: total antioxidant capacity; SOD: superoxide dismutase; MDA: malondialdehyde; PI3K-Akt: phosphoinositide 3-kinase- protein kinase; AMPK: adenosine monophosphate-activated protein kinase; ATIC/GPAT: IMP cyclohydrolase/glutamine-PRPP amidotransferase; SCFAs: short-chain fatty acids; IMF: intramuscular fat; FASN: fatty acid synthase; PPARγ: peroxisome proliferator-activated receptor-γ; FABP4: fatty acid-binding protein 4; C/EBPα: CCAAT/enhancer-binding protein α; AMPK-PGC1α: AMPK-peroxisome proliferator-activated receptorγ coactivator 1-α; MyHC1: myosin heavy chain 1; MyHC4: myosin heavy chain 4; MyHC2: myosin heavy chain 2; MyHC7: myosin heavy chain 7

Several recent studies have demonstrated that feed fermentation utilizing various probiotic microorganisms, including *Bacillus subtilis* [17, 126, 143], *Enterococcus faecium* [17, 126], and *Clostridium butyricum* [143], effectively improve pork quality. This improvement may be attributed to the pivotal role of probiotics and their metabolites in regulating meat quality. According to the existing research, we speculate that fermented feed may improve meat quality by modulating the gut microbiota and optimizing the composition of short-chain fatty acids (SCFAs) in the intestine (Fig. 3). Previous studies reveled that gut microbiota may regulate the lipid metabolism and utilized the specific gut microbial community from Laiwu pigs providing a promising strategy for ameliorating the meat quality of commercial pigs [144, 145]. Furthermore, the abundance of intestinal flora is associated with the accumulation of fatty acids. Correlations between intestinal flora and fatty acids showed that *Clostridium_sensu_stricto_1* promoted the accumulation of PUFA and n-6 PUFA while inhibiting MUFA deposition. Similar correlations were also observed in *Terrisporobacter* and *Lachnospiracea*e [107]. Moreover, intestinal microbiota is related to IMF content, and improving IMF content through modulating gut microbiota is an effective strategy [146]. Qi et al. [5] identified positive correlations between *Clostridium* abundance and IMF deposition, while health-related bacteria, such as *Butyricicoccus*, *Eubacterium*, *Phascolarctobacterium*, and *Oribacterium*, showed negative associations with abdominal fat area and myofiber density. In addition to IMF content, the microbial community has strong correlations with the composition of volatile compounds. Liu et al. [126] revealed that fermented feed supplementation increased the relative abundance of *Phascolarctobacterium*, *Faecalibacterium*, and *UCG_002*, while decreasing *UCG-010_unclassified*. Further correlation analysis showed that *Phascolarctobacterium* and *Faecalibacterium* exhibited positive correlations with total aldehydes and IMP content, whereas *UCG-010_unclassified* showed negative associations with these flavor compounds. Furthermore, SCFAs, as microbial fermentation products, may directly regulate the expression of genes related to lipid metabolism or act as signal molecules to influence the IMF deposition [107]. Taken together, fermented feed shapes the microbiota and provides some beneficial metabolites. These microorganisms and metabolites, in turn, exert a positive effect on the meat quality of the organism. However, it is worth noting that research on this aspect is not deep enough, and further investigations are warranted to elucidate the exact mechanism by which fermented feed regulates the quality of pork by affecting the gut microbiota.

### Regulates lipid metabolism

Many researchers have confirmed that fermented feed can enhance pork quality by regulating lipid metabolism (Fig. 3). Previous studies have demonstrated that dietary supplementation with fermented feed upregulated the expression of genes regarding fatty acid synthesis, uptake and transport, such as fatty acid synthase (*FASN*), CCAAT/enhancer-binding protein α (*C/EBPα*), peroxisome proliferator-activated receptor-γ (*PPARγ*), stearoyl-CoA desaturase enzyme 1 (*SREBP1*), and fatty acid-binding protein 4 (*FABP4*) [17, 89, 102, 103]. Our preliminary research results indicated that the growth arrest and DNA damage 45A (GADD45a) as a novel regulator to promote fat deposition and inhibit muscle regeneration and mitochondrial function. This effect is mediated through its binding interaction with and subsequent promotion of the degradation of mitochondrial complex protein ATP synthase F1 subunit alpha (ATP5A1) [147, 148]. Further research revealed that fermented feed upregulated the expression of GADD45a, suggesting that fermented feed may increase the percentage of TAG in muscle by regulating GADD45a, thereby affecting pork quality. Moreover, the inclusion of fermented feed has been demonstrated to favorably modify the lipid profile of the *longissimus thoracis* muscle, ultimately ameliorating pork quality. Specifically, a 10% supplementation of fermented feed was found to reduce the level of phosphatidylcholine (PC) (33:0e), a lipid molecule strongly positively correlated with shear force and negatively correlated with the mRNA expression of myosin heavy chain-I (*MyHC-*I) and myosin heavy chain-IIx (*MyHC-IIx*) [139]. Furthermore, lipidomics analysis indicates that fermented liquid feed supplementation elevates the concentrations of MUFA and PUFA in pork while reducing the n-6:n-3 ratio [103]. These findings suggest that fermented feed improves meat quality by modulating lipid metabolism, particularly through alterations in glycerophospholipid pathways.

### Promotes the conversion of fast-twitch fibers to slow-twitch fibers

Skeletal fibers are classified into four distinct types based on the main expressed MyHC subtypes: I, IIa, IIx, and IIb. Type I fibers (slow-twitch, oxidative) characterized by greater mitochondria and oxidative enzyme activity, primarily utilize lipids as an energy substrate. Type IIb fibers (fast-twitch, glycolytic) rely predominantly on glycolytic metabolism, driving energy from glycogen and glucose, whereas type IIa (fast-twitch, oxidative) and type IIx (fast-twitch, oxido-glycolytic) exhibit intermediate metabolic properties between type I and IIb fibers [149]. Different skeletal muscle fiber subtypes exhibit distinct metabolic and physiological properties and are therefore closely linked with color, water-holding capacity, marbling, pH, and other meat quality parameters [150, 151]. It is generally accepted that a high proportion of type I and IIa fibers, coupled with a reduction in type IIx and IIb fibers, is generally associated with superior meat quality [152]. Although the evolution process has determined the number of skeletal muscle fibers in animals to be constant, the composition of muscle fiber types can be regulated in response to nutritional interventions [149, 153–155]. Numerous studies have indicated that fermented feed supplementation promotes a shift from fast-twitch fibers to slow-twitch fibers (Fig. 3). For example, Li et al. [105] indicated that dietary supplementation with fermented Inonotus obliquus enhanced the expression level of *MyHC *I and myosin heavy chain-IIa (*MyHC *II*a*), while decreasing the expression level of myosin heavy chain-IIb (*MyHC *II*b*). Xie et al. [89] reported that dietary supplementation of finishing pigs with fermented soybean meal upregulated the expression levels of the *MyHC-*I and *MyHC-*II*a* in the *longissimus thoracis*. Qiu et al. [21] also suggested that a fermented diet led to higher levels of *MyHC-*I. Fermented liquid feed has been shown to increase the proportion of slow-twitch muscle fibers while reducing the proportion of fast-twitch muscle fibers in pork, thereby contributing to improved meat quality [103]. Similarly, a recent study demonstrated that fermented feed increased the proportion of slow-twitch fibers and promoted skeletal fiber switching from glycolytic type II to oxidative type I. Further analysis showed that fermented feed facilitated this conversion probably by activating the AMPK-peroxisome proliferator-activated receptorγ coactivator 1-α (AMPK/PGC1α) signaling pathway. However, it remains unclear that the specific molecular mechanism how fermented feed induces the conversion of fast-twitch fibers to slow-twitch fibers via the AMPK/PGC1α pathway [139]. Further studies should be conducted to explore whether any beneficial metabolites produced by probiotics in fermented feed could regulate the muscle fiber switching process.

### Enhances antioxidant capacity

Oxidation is one of the major causes for quality deterioration during the processing and storage of meat, which may exert adverse effects on some meat quality traits such as meat color, water-holding capacity, tenderness, and nutrient value. Oxidation of oxymyoglobin to methemoglobin results in brown or discolored lean meat, while lipid oxidation leads to the production of off-flavor and decreases the nutritional values of meat [156]. Some researchers have emphasized that pork shows high contents of endogenous antioxidant compounds exhibiting a positive association with meat quality. Generally, the higher the activity of enzymatic antioxidants in muscle, the lower the content of the peroxide product malondialdehyde (MDA), the greater the muscle water holding capacity, the brighter the meat color and the more tender the meat [157, 158]. As illustrated in Fig. 3, fermented feed effectively enhances the activity of enzymatic antioxidants, ultimately ameliorating the pork quality. A recent study performed by Li et al. [105] reported that supplementation with 8 g/kg Inonotus obliquus fermented for 7 d increased total-superoxide dismutase (T-AOC), superoxide dismutase (SOD) and catalase (CAT) activities in muscle, accompanied by upregulated expression of antioxidant-related genes. Zhao et al. [106] observed an increase in T-AOC in the *longissimus thoracis* after feeding with fermented broccoli residues. A study conducted by Liu et al. [139] suggested that SOD and CAT activities in the muscle were increased after supplementing with *Bacillus subtilis* and *Enterococcus faecium* co-fermented feed. Hao et al. [24] revealed that greater SOD and glutathione peroxidase (GSH-Px) activities were observed in porcine *longissimus muscle* with 8% fermented feed inclusion. Tian et al. [19] showed an increase of SOD and GSH-Px activities in the *longissimus thoracis* muscle after supplementing with fermented okara. Similar results were obtained by Xie et al. [89], who reported that pigs fed with fermented soybean meal diet increased T-AOC, but decreased MDA content in *longissimus thoracis*. These findings verified that fermented feed supplementation can effectively increase the activity of antioxidants in muscle, including GSH-Px, T-AOC, SOD, and CAT, suggesting that the improvement of pork quality can be partly explained by an increase in antioxidant activity in muscle.

## Conclusions and perspectives

In summary, fermented feed, as a functional feed, has brought many beneficial effects to livestock production. Microbial fermentation enables the utilization of unconventional feed materials, such as yellow wine lees, paper mulberry, tea residues, navel orange pulp, okara, and bamboo powder in swine production, while simultaneously enhancing the nutritional profiles of feedstuffs and positively influencing pork quality. In recent years, researchers have reported that fermented feed serves as an effective nutritional intervention for improving pork quality, but there are still several critical challenges that need further investigation. First of all, substantial variability exists in product quality across different microbial strains and fermentation conditions. Future studies should prioritize strain optimization through metagenomic analysis of gut microbiota in indigenous swine breeds, facilitating the identification of high-efficiency strains and the establishment of a specialized probiotic database based on fermentation performance metrics. Second, the manufacturing techniques for fermented feed necessitate further optimization. Multi-strain and microbial-enzyme synergistic fermentation demonstrates considerable efficacy in degrading anti-nutritional factors and enhancing nutrient bioavailability, warranting broader application in swine production. Third, although unconventional feed ingredients offer economic and nutritional advantages, their supplementation levels must be rigorously controlled, as excessive amounts may adversely affect growth performance and meat quality [20, 30, 109]. Additionally, more efforts are needed to expand and optimize the development and utilization of novel unconventional feed resources. Fourth, the lack of standardized evaluation systems leads to considerable inconsistency in fermented product quality. The development of industry-wide standards covering fermentation strains, fermentation process, storage and transportation, and quality assessment is crucial to ensure product uniformity and safety. Fifth, the underlying mechanisms by which fermented feed regulates pork quality remain incompletely elucidated. Further investigations and explorations are needed to clarify its effects on lipid metabolism, gut microbiota, and flavor compounds formation at the molecular level.

## Data Availability

No datasets were generated or analysed during the current study.

## Abbreviations

AA: Amino acid
ADF: Acid detergent fiber
AMP: Adenosine monophosphate
AMPK: Adenosine monophosphate-activated protein kinase
AMPK/PGC1α: AMPK-peroxisome proliferator-activated receptorγ coactivator 1-α
ATIC/GPAT: IMP cyclohydrolase/glutamine-PRPP amidotransferase
ATP5A1: ATP synthase F1 subunit alpha
CAT: Catalase
C/EBPα: CCAAT/enhancer-binding protein α
CF: Crude fiber
CLA: Conjugated linoleic acid
CP: Crude protein
DM: Dry matter
EAA: Essential amino acids
EE: Ether extract
FAA: Flavor amino acids
FABP4: Fatty acid-binding protein 4
FASN: Fatty acid synthase
GADD45a: Growth arrest and DNA damage
GSH-Px: Glutathione peroxidase
HMP: 2-Hydroxy-4-methylpentanoic acid
IBD: Inflammatory bowel disease
IMF: Intramuscular fat
IMP: Inosine monophosphate
LAB: Lactic acid bacteria
MDA: Malondialdehyde
MUFA: Monounsaturated fatty acids
MyHC-I: Myosin heavy chain-I
MyHC-IIa: Myosin heavy chain-IIa
MyHC-IIx: Myosin heavy chain-IIx
MyHC-IIb: Myosin heavy chain-IIb
NDF: Neutral detergent fiber
NEAA: Nonessential amino acids
n-3 PUFA: n-3 polyunsaturated fatty acids
NSP: Non-starch polysaccharide
PI3K-Akt: Phosphoinositide 3-kinase- protein kinase
PPARγ: Peroxisome proliferator-activated receptor-γ
PUFA: Polyunsaturated fatty acids
SCFAs: Short-chain fatty acids
SFA: Saturated fatty acids
SOD: Superoxide dismutase
SREBP1: Stearoyl-CoA desaturase enzyme 1
SSF: Solid-state fermentation
TAG: Triglycerides
T-AOC: Total superoxide dismutase
TCA-SP: Trichloroacetic acid soluble protein
UFA: Unsaturated fatty acids
